# Functional, Cognitive, Physical, and Vascular Outcomes 9 Years After Lacunar and Mild Cortical Ischemic Stroke

**DOI:** 10.1212/WNL.0000000000214018

**Published:** 2025-08-20

**Authors:** Daniela Jaime Garcia, Stephen D.J. Makin, Caroline A. McHutchison, Vera Cvoro, Maria Del C. Valdés Hernández, Eleni Sakka, Francesca M. Chappell, Fergus Doubal, Joanna M. Wardlaw

**Affiliations:** 1 Centre for Clinical Brain Sciences, School of Clinical Sciences, College of Medicine and Veterinary Medicine, The University of Edinburgh, United Kingdom;; 2 UK Dementia Research Institute at the University of Edinburgh, United Kingdom;; 3 Medical Sciences and Nutrition, School of Medicine, University of Aberdeen, United Kingdom;; 4 Department of Psychology, School of Philosophy, Psychology, and Language Sciences, The University of Edinburgh, United Kingdom; and; 5 NHS Fife, Victoria Hospital, Kirkcaldy, United Kingdom.

## Abstract

**Background and Objectives:**

Data on outcomes after minor stroke, especially lacunar stroke, are lacking or based on short follow-up. Outcome differences between lacunar and cortical stroke remain unclear but may provide insights into small vessel disease (SVD) etiology and prognosis. We report on dementia, death, recurrent stroke, and function up to 9 years after lacunar or minor cortical ischemic stroke.

**Methods:**

In this 9-year longitudinal observational cohort study, we prospectively recruited participants presenting with mild, nondisabling lacunar or cortical ischemic stroke to Lothian Stroke Services in Edinburgh, United Kingdom. At baseline, we collected data on sociodemographics, vascular risk factors, dependence, cognition, and brain MRI. At 9 years, we used questionnaires (including items from the Stroke Impact Scale) and hospital/general practitioner records to assess dementia, recurrent stroke, death, cardiac diseases, vascular risk factors, dependence, recovery, and functionality.

**Results:**

We recruited 264 participants; clinical data were available for 243 participants (92%) (baseline mean age 67 years (SD 12), 42% female, 44% with lacunar stroke) and self-reported functional data for 96 (36%) at a mean follow-up of 8.5 years (SD 0.57). Dementia was diagnosed in 9.4% of participants with lacunar stroke and 12.4% of those with cortical stroke, with risk increasing with age (odds ratio [OR] 1.08; CI 1.030–1.130). Recurrent stroke occurred in one-third of all participants; risk increased with the presence of vascular risk factors (OR 2.27; CI 1.287–4.032). Participants with cortical stroke were more likely to die compared with those with lacunar stroke (χ^2^ = 8.2; *p* = 0.004). Age (OR 1.09; CI 1.051–1.133), male sex (female OR 0.40, CI 0.196–0.818), and white matter hyperintensities (OR 1.36; CI 1.112–1.664) increased risk of death. Moderate/severe disability was reported by 12% of participants and cognitive concerns by 49%–55%. Affected limb recovery and balance were worse after lacunar stroke (*W* = 705.5, *p* = 0.00).

**Discussion:**

Long-term outcomes after minor stroke were frequently suboptimal and varied by stroke subtype, highlighting the lasting impact of both lacunar and mild cortical ischemic strokes on quality of life. Improving long-term outcomes after mild stroke remains an important target for refining clinical care and shaping future research directions.

## Introduction

Cerebral small vessel disease (SVD) is a leading cause of vascular dementia and cognitive impairment and is associated with lacunar or hemorrhagic stroke, mobility problems, and neuropsychiatric symptoms.^1-5^ Lacunar strokes account for a quarter of all ischemic strokes and are distinctive in their symptomatology.^6^ Lacunar infarcts are small (<15 mm in diameter), occur in subcortical regions, and result from disease of a single perforating artery and intrinsic small vessel pathology.^7^

Compared with other stroke subtypes, lacunar stroke is generally considered to have a favorable prognosis, characterized by small infarct size, minimal risk of early death, low early recurrent stroke rates, and typically nondisabling effects in the initial months after stroke. However, the long-term prognosis of lacunar infarction remains less well characterized compared with other stroke subtypes.

Research on lacunar stroke outcomes is limited, with most studies being small with short follow-up periods, rarely extending beyond 4 years.^8^ Most studies lack patient-reported measures on well-being and disability. It is important to consider prognosis within a broader context, particularly because lacunar strokes tend to occur at slightly younger ages, are associated with high survival rates, and frequently involve cognitive impairment.^9^ Patients with SVD have expressed concerns regarding their long-term prognosis, highlighting a lack of information from health care providers.^9^ Longitudinal data on the outcomes of lacunar stroke, and their comparison with similarly mild cortical ischemic stroke, can offer valuable insights to help guide clinical management and inform research priorities.

In this study, we report long-term clinical outcomes, including dementia, recurrent stroke, death, and participant-reported functional and cognitive outcomes, in a prospective study of participants with lacunar ischemic stroke and those with similarly mild cortical ischemic stroke at 9 years after stroke.

## Methods

### Participants and Study Design

We prospectively recruited patients presenting with acute minor ischemic stroke to Lothian Stroke Services in Edinburgh, United Kingdom, between May 2010 and May 2012. Based on clinical assessment by the duty stroke registrar, patients were either admitted to the stroke unit or managed in the outpatient stroke clinic. Recruitment occurred through several pathways, including direct identification by the clinical research fellow, referrals from treating clinicians, or input from the stroke research nurse. Protocol details have been described previously.^10,11^ Minor ischemic stroke was defined as a focal onset of neurologic symptoms lasting >24 hours with no alternative explanation, a NIH Stroke Scale (NIHSS) score <8, and an expected outcome of independence (modified Rankin Scale [mRS] score <3). The expected outcome was determined by the recruiting clinician based on the initial clinical assessment and estimated prognosis. All patients were dementia free at recruitment and received secondary stroke prevention treatment in line with UK clinical guidelines. We excluded patients who did not have a confirmed stroke diagnosis, those whose symptoms resolved within 24 hours, those who were unable to consent, those who had a disabling stroke, those who had a medical condition with a life expectancy of less than 1 year, or those who had contraindications to MRI.

### Standard Protocol Approvals, Registrations, and Participant Consents

All participants provided written informed consent. This study was approved by Lothian Ethics Medical Research Committee (REC 09/81,101/54) and NHS Lothian R&D Office (2009/W/NEU/14).

### Baseline Assessments and 1-Year, 3-Year, and 9-Year Follow-Ups

At baseline, we assessed sociodemographic/lifestyle variables, vascular risk factors, NIHSS score, functional dependency (mRS score), cognition (Addenbrooke Cognitive Examination–Revised [ACE-R] score),^12^ premorbid intelligence (National Adult Reading Test score),^13^ ischemic heart disease (IHD) diagnosis, and brain MRI. Vascular risk factors included hypertension (defined as blood pressure ≥140/90 mm Hg at presentation or a previous diagnosis), hypercholesterolemia (defined as cholesterol >5 mmol/L at presentation or a previous diagnosis), diabetes mellitus (diagnosed on admission or a previous diagnosis in accordance with the World Health Organization criteria), and atrial fibrillation. Cognitive testing began after the first 56 participants because of ethics approval delays.

Participants were invited for a follow-up visit at 1 and 3 years after stroke, including face-to-face assessments when possible and a review of medical records.^11^ We collected mRS and ACE-R scores and data on recurrent vascular events or any further clinical events of note, including recurrent stroke, TIA, intracranial hemorrhage (ICH), myocardial infarction, IHD, and new diagnosis of vascular risk factors since the previous visit. When a participant was unable to attend an in-person study visit, home visits or telephone interviews were offered. Otherwise, information was sought from general practitioner (GP) and centralized medical records. In cases where participants had died, cause of death was obtained from hospital records and death certificates.

At 9 years, we used multiple overlapping ascertainment methods, including central medical records and self-reported and GP-derived data, to assess dementia, recurrent stroke incidence, deaths, cardiac events/diagnoses, and new vascular risk factor diagnoses. Dementia subtype data were not collected because of inconsistent availability and reliability. We sent a questionnaire to living participants to assess disability and dependence using the mRS and evaluate several aspects of function, cognition, self-reported well-being, mobility, dexterity, stroke recovery, and activities of daily living using items from the Stroke Impact Scale.^14^ We also collected data on participants' current living arrangement.

### Imaging and Stroke Diagnosis

At baseline, participants underwent brain MRI on a 1.5T scanner (Signa LX; General Electric, Milwaukee, WI). The index infarct was classified as a recent small subcortical infarct (RSSI) or a cortical infarct based on the size, location, shape, and age in relation to stroke presentation. Stroke diagnosis was confirmed and subtyped as either “cortical” or “lacunar” using the Oxfordshire Community Stroke Project Classification by an expert panel of neurologists, stroke physicians, and neuroradiologists based on the clinical stroke syndrome and MRI scans.^15^ RSSIs were defined as signal characteristics consistent with a recent infarct in the deep gray or white matter of the cerebral hemispheres or brainstem with an axial diameter of <20 mm. Cortical infarcts were characterized as signal characteristics consistent with a recent infarct involving the cortex or large subcortical infarcts with an axial diameter of >20 mm.

### MRI Processing

Intracranial volumes (ICVs) were computationally generated, checked for accuracy, and edited manually when required. White matter hyperintensity (WMH) volumes were extracted semiautomatically from co-registered fluid-attenuated inversion recovery and T2*-weighted sequences using minimum variance quantization in the fused image pairs.^16^ Index, acute, old, and incident infarcts were manually segmented and excluded from total WMH volumes by an experienced image analyst. An experienced neuroradiologist (J.M.W.) assessed scans for index and previous infarcts or hemorrhages, WMHs, lacunes, perivascular spaces, microbleeds, and atrophy and calculated the summary SVD scores (assigning 1 point for the following: (1) a Fazekas score of 3 in the periventricular white matter or ≥2 in the deep white matter; (2) lacunes present; (3) microbleeds present; (4) >10 counts of enlarged perivascular spaces in the basal ganglia) according to STRIVE criteria and using validated scales.^17,18^

### Statistical Analysis

Analyses were performed using R (version 4.4.1). We expressed WMH volumes as % ICV to adjust for variations in head size. WMH volumes as % ICV were log-transformed to improve model fit. To reduce the number of predictors and avoid model overfitting, we assigned equal weight to hypertension, hypercholesterolemia, diabetes, and smoking history (current smoker or ex-smoker within 1 year) in the calculation of a baseline vascular risk factor composite score.^19,20^

We compared continuous variables between groups with independent *t* tests (reporting 95% CIs and *p* values) and categorical variables with χ^2^ tests (reporting χ^2^ and *p* values). For ordinal or non-normally distributed variables, Mann-Whitney *U* tests were applied, reporting the *W* statistic and *p* values.

Logistic regression was used to examine the relationship between stroke subtype and dementia, recurrent stroke, death, and cardiovascular events. Models were adjusted for age, sex, vascular risk factor composite score, and baseline WMH volume. Logistic regression models with recurrent stroke and death as outcome variables were further adjusted for baseline NIHSS scores. The *rms* package (R, version 6.9-0) was used to assess the discrimination and calibration of logistic regression models by evaluating model performance, including using the *c*-statistic (area under the curve) for discrimination and calibration curves to assess alignment between predicted outcomes and observed outcomes.

To account for deceased participants, separate regression models were implemented at 0–1, 1–3, and 3–9 years for recurrent stroke outcomes. Kaplan-Meier survival curves and log-rank tests were performed using the *survival* (R, version 3.2-13) and *survminer* (R, version 0.4.9) packages. Significance was defined as *p* < 0.05.

### Data Availability

Anonymized data not published within this article can be made available by request to the senior author of this publication from any qualified investigator.

## Results

**Figure 1:**
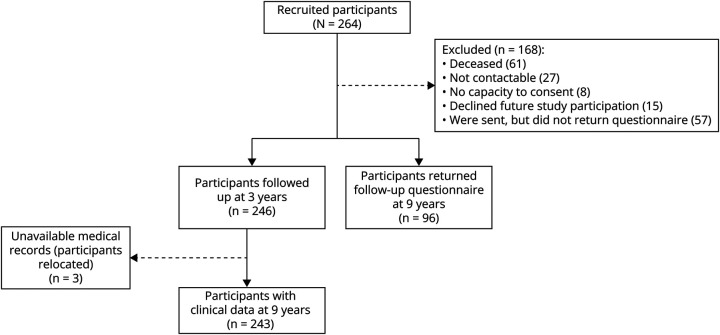
Participant Flowchart Participant flowchart for clinical outcomes (left) based on a combination of general practitioner records, hospital record data, and participant questionnaire response and for functional and self-reported well-being outcomes (right) based on a postal questionnaire sent to participants 9 years after initial study recruitment.

We recruited 264 participants (mean age 66.9 years [SD 11.8], 41.7% female, 44.7% with lacunar) within 3 months of a mild, nondisabling lacunar or cortical ischemic stroke. Clinical outcome data were available for 264 (100%) at 1 year, 246 (93%) at 3 years (16 declined, 2 relocated), and 243 (92%) at 9 years (3 relocated; mean follow-up time 8.5 years [SD 0.57, range 7.4–9.3]) (Figure 1). In addition, we sent questionnaires to 153 alive participants (111 excluded: 61 deceased, 27 uncontactable, 15 declined, 8 lacked capacity to consent) and received 96 (62.75%) (mean age of responders 71 years [SD 10], 40% female, 46.9% with lacunar) (Figure 1).

Among participants with 9-year outcome data (n = 243), the mean baseline age was 67 years (SD 12), 42% were female, and the stroke subtype was lacunar in 43.6%. Baseline cohort characteristics are summarized in Table 1. Participants with lacunar stroke were slightly younger (65 years [SD 11] vs 68 years [SD 12]; *p* = 0.03), had higher summary SVD scores (2.53 [SD 1.25] vs 2.12 [SD 1.15]; *p* = 0.01), and had higher median NIHSS scores (1 [IQR 0–2] vs 0 [IQR 0–1]; *p* = 0.01) compared to those with cortical stroke.

**Table 1:** Baseline Cohort Characteristics

	Lacunar (N = 106)	Cortical (N = 137)	Overall (N = 243)	Result (*p* value)
Age, y, mean ± SD	65 ± 11	68 ± 12	67 ± 12	0.249, 6.158 (0.03)^a^
Sex, female, n (%)	46 (43)	55 (40)	101 (42)	0.143 (0.70)^b^
mRS score, n (%)				7,333.5 (0.88)^c^
No symptoms at all	17 (16)	28 (20)	45 (19)	—
No significant disability	49 (46)	51 (37)	100 (41)	—
Slight disability	19 (18)	26 (19)	45 (19)	—
Moderate disability	16 (15)	28 (20)	44 (18)	—
Moderate-severe disability	4 (4)	4 (3)	8 (3)	—
Bedridden	1 (1)	0 (0)	1 (0)	—
NIHSS score, median (IQR)	1 (0–2)	0 (0–1)	1 (0–2)	5,875 (0.007)^c^
Diabetes, yes, n (%)	11 (10)	17 (12)	28 (12)	0.083 (0.77)^b^
Hypertension, yes, n (%)	76 (72)	101 (74)	177 (73)	0.04 (0.83)^b^
Hypercholesterolemia, yes, n (%)	66 (62)	82 (60)	148 (61)	0.06 (0.80)^b^
Atrial fibrillation, yes, n (%)	6 (6)	14 (10)	20 (8)	1.09 (0.23)^b^
Current smoker or ex-smoker <1 y ago, n (%)	46 (43)	47 (34)	93 (38)	1.723 (0.18)^b^
NART score, mean ± SD	34 ± 10	35 ± 10	35 ± 10	−2.216, 4.690 (0.47)^a^
Missing, n (%)	44 (41.5)	55 (40.1)	99 (40.7)	—
ACE-R score, mean ± SD	87 ± 7.8	88 ± 8.6	88 ± 8.2	−2.399, 2.962 (0.83)^a^
Missing, n (%)	43 (40.6)	53 (38.7)	96 (39.5)	—
Summary SVD score, median (IQR)	2 (1–3)	2 (1–3)	2 (1–3)	−0.719, −0.103 (0.01)^a^
WMH volume, mm^3^ as % ICV, mean ± SD	1.6 ± 1.7	1.5 ± 1.6	1.5 ± 1.7	−0.579, 0.273 (0.47)^a^

Abbreviations: ACE-R = Addenbrooke Cognitive Examination–Revised; ICV = intracranial volume; mRS = modified Rankin Scale; NART = National Adult Reading Test; NIHSS = NIH Stroke Scale; SVD = small vessel disease; WMH = white matter hyperintensity.

a Independent-samples *t* test (95% CI).

b Chi-square (χ^2^).

c Mann-Whitney *U* test (*W*).

Participants with clinical outcome data at 9 years had worse cognition at baseline compared with those without (ACE-R score: 88 [SD 8.2] vs 95 [SD 3.3]; *p* ≤ 0.001) (eTable 1). Participants who returned the outcome questionnaire were younger at baseline than those who did not (65 years [SD 11] vs 68 years [SD 12]; *p* = 0.02) and performed better on cognitive assessments (ACE-R score: 90 [SD 6.5] vs 87 [SD 8.8]; *p* = 0.003), had lower summary SVD scores (2.29 [SD 1.21] vs 2.66 [SD 1.06]; *p* ≤ 0.001), and had lower WMH volumes (1 mm^3^ [SD 1.1] vs 1.9 [SD 1.8]; *p* ≤ 0.001) at baseline (eTable 2).

### Clinical Outcomes: Dementia, Recurrent Stroke, Death, Cardiac Disease, and Vascular Risk Factors

#### Dementia

**Figure 2:**
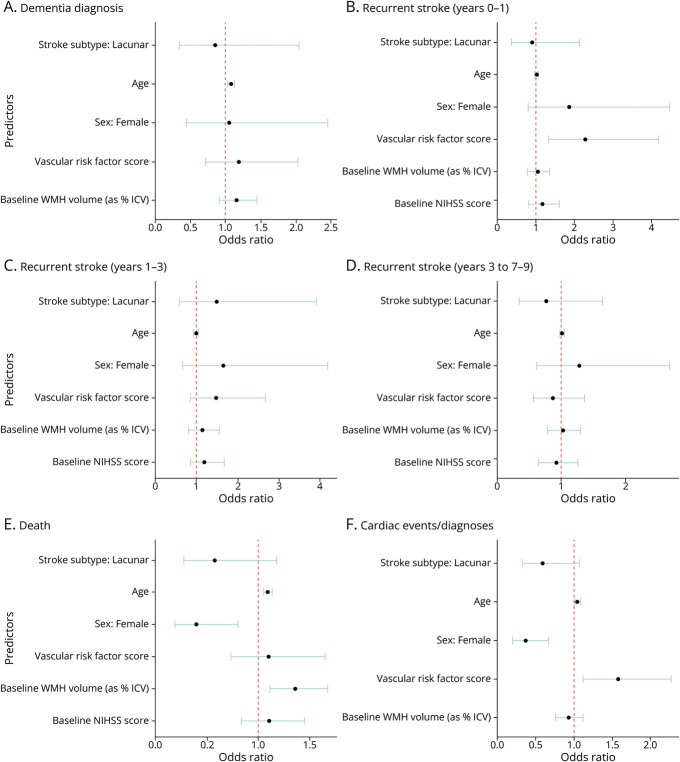
Stroke Subtype and Clinical Outcomes at 9 Years Logistic regression results for the association between possible predictors (including stroke subtype) and clinical outcomes: dementia, recurrent stroke, death, and cardiac diseases. ICV = intracranial volume; NIHSS = NIH Stroke Scale; WMH = white matter hyperintensity.

Between baseline and 9-year follow-up, 9.4% (n = 10/106) of participants with lacunar stroke and 12.4% (n = 17/137) of those with cortical stroke had a dementia diagnosis. Age was significantly associated with dementia risk (odds ratio [OR] 1.08, 95% CI 1.030–1.130) (Table 2 and Figure 2).

**Table 2:** Stroke Subtype and Clinical Outcomes at 9 Years After Lacunar or Mild Cortical Ischemic Stroke

Predictor	OR	95% CI	*p* Value	*c*-statistic
Dementia diagnosis (n = 243)				
Stroke subtype (lacunar)	0.85	0.352–2.080	0.73	0.76
Age	1.08	1.030–1.130	0.001	—
Sex (female)	1.05	0.448–2.473	0.90	—
Vascular risk factor score	1.19	0.713–2.014	0.49	—
Baseline WMH volume as % ICV	1.15	0.922–1.456	0.20	—
Recurrent stroke, years 0–1 (n = 232)				0.70
Stroke subtype (lacunar)	0.89	0.371–2.147	0.80	—
Age	1.01	0.970–1.055	0.57	—
Sex (female)	1.87	0.801–4.385	0.14	—
Vascular risk factor score	2.27	1.287–4.032	0.004	—
Baseline WMH volume as % ICV	1.04	0.792–1.365	0.77	—
Baseline NIHSS score	1.16	0.835–1.631	0.36	—
Recurrent stroke, years 1–3 (n = 220)				0.67
Stroke subtype (lacunar)	1.49	0.581–3.854	0.40	—
Age	0.98	0.938–1.035	0.56	—
Sex (female)	1.64	0.661–4.101	0.28	—
Vascular risk factor score	1.48	0.837–2.618	0.17	—
Baseline WMH volume as % ICV	1.14	0.835–1.569	0.39	—
Baseline NIHSS score	1.20	0.865–1.675	0.27	—
Recurrent stroke, years 3–9 (n = 220)				0.59
Stroke subtype (lacunar)	0.76	0.349–1.665	0.49	—
Age	1.01	0.980–1.050	0.39	—
Sex (female)	1.28	0.615–2.699	0.50	—
Vascular risk factor score	0.87	0.568–1.360	0.56	—
Baseline WMH volume as % ICV	1.02	0.799–1.312	0.84	—
Baseline NIHSS score	0.92	0.670–1.28	0.64	—
Death (n = 243)				0.81
Stroke subtype (lacunar)	0.58	0.284–1.185	0.13	—
Age	1.09	1.051–1.133	<0.001	—
Sex (female)	0.40	0.196–0.818	0.01	—
Vascular risk factor score	1.09	0.734–1.642	0.64	—
Baseline WMH volume as % ICV	1.36	1.112–1.664	0.002	—
Baseline NIHSS score	1.10	0.838–1.452	0.48	—
Cardiac diseases^a^ (n = 243)				0.70
Stroke subtype (lacunar)	0.59	0.329–1.079	0.08	—
Age	1.04	1.019–1.081	0.001	—
Sex (female)	0.36	0.201–0.676	0.001	—
Vascular risk factor score	1.57	1.111–2.236	0.01	—
Baseline WMH volume as % ICV	0.92	0.769–1.119	0.43	—

Abbreviations: ICV = intracranial volume; NIHSS = NIH Stroke Scale; OR = odds ratio; WMH = white matter hyperintensity.

Logistic regression results for the association between dementia, recurrent stroke, death, and cardiac disease and possible predictors, including stroke subtype, age, sex, vascular risk factors, baseline WMH volume, and NIHSS score where appropriate.

a Cardiac disease diagnoses include ischemic heart disease/myocardial infarction (n = 67), heart failure (n = 8), pacemaker implantation (n = 1), supraventricular tachycardia (n = 1), cardio-ablation (n = 1), left ventricular hypertrophy (n = 1), cardiovascular disease listed on the death certificate/medical record (not specified) (n = 2), aortic valve replacement (n = 2), percutaneous coronary intervention/transcatheter aortic valve implantation (n = 1), and fenestrated endovascular aortic repair (n = 1).

#### Recurrent Stroke

Between baseline and 9-year follow-up, 31.7% (n = 77/243) of participants had at least 1 recurrent stroke, including 31.1% of participants with lacunar stroke (n = 33/106) and 32.1% (n = 44/137) of those with cortical stroke. Of these, 66 had 1, 8 had 2, and 3 had 3 recurrent strokes.

Within the first year after stroke, 10.8% (n = 11/102) of participants with lacunar stroke and 11.5% (n = 15/130) of those with cortical stroke had at least 1 recurrent stroke. Between 1 and 3 years, 14.8% (n = 12/81) of participants with lacunar stroke and 9.3% (n = 10/108) of those with cortical stroke had at least 1 recurrent stroke. Between 3 and 9 years, 13% (n = 13/100) of participants with lacunar stroke and 17.5% (n = 21/120) of those with cortical stroke had at least 1 recurrent stroke (Table 2 and Figure 2 and eFigure 1). Vascular risk factor score was independently associated with increased risk of recurrent stroke, although only within the first year after index stroke (OR 2.27, 95% CI 1.287–4.032) (Table 2 and Figure 2).

In addition, between baseline and 9 years, 3.8% (n = 4/106) of participants with lacunar stroke and 4.4% (n = 6/137) of those with cortical stroke had at least 1 TIA. ICH occurred in 1.9% (n = 2/106) of participants with lacunar stroke and 1.5% (n = 2/137) of those with cortical stroke.

#### Deaths

**Figure 3:**
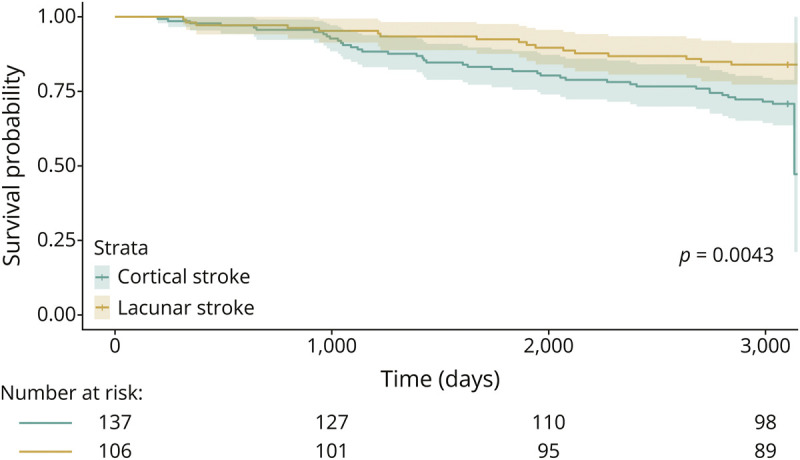
Kaplan-Meier Survival Curves Comparing Death Rates Between Cortical and Lacunar Stroke at 9 Years After Stroke (n = 143) The log-rank test indicated a significant difference in survival probabilities between the groups, where cortical stroke may have worse survival outcomes compared with lacunar stroke (χ^2^ = 8.2; *p* = 0.004).

26.3% (64/243) of participants died, including 19.8% (n = 21/106) of those with lacunar stroke and 31.3% (n = 43/137) of those with cortical stroke. The average age at death was 79.3 years (SD 10.3) in those with cortical stroke and 80.5 years (SD 8) in those with lacunar stroke. Age (OR 1.09, 95% CI 1.051–1.133), male sex (female sex OR 0.40, 95% CI 0.196–0.818), and baseline WMH volume (OR 1.36, 95% CI 1.112–1.664) were significantly associated with death (Table 2 and Figure 2). Participants with cortical stroke had worse survival outcomes than those with lacunar stroke (Kaplan-Meier curve and log-rank test on Figure 3; χ^2^ = 8.2; *p* = 0.004).

In the lacunar stroke group, malignancies were the leading cause of death (38.1%, n = 8/21), followed by other/unknown causes (23.8%, n = 5/21), recurrent stroke or complications from stroke (14.3%, n = 3/21), and pulmonary/respiratory issues (4.8%, n = 1/21). In the cortical stroke group, the most common primary cause of death was vascular or cardiovascular disease (27.9%, n = 12/43), followed by malignancies (25.6%, n = 11/43), recurrent stroke or complications from stroke (18.6%, n = 8/43), and pulmonary/respiratory issues or other/unknown causes (14%, n = 6/43 each).

#### Cardiac Diseases

Cardiac disease diagnoses, including IHD, heart failure, and arrhythmias, were more common in participants with cortical stroke (40% [n = 55/137]) than in those with lacunar stroke (26.4% [n = 28/106]).

IHD was reported in 22.6% (n = 24/106) of participants with lacunar stroke and 31.4% (n = 43/137) of those with cortical stroke. Heart failure occurred in 1.9% (n = 2/106) of participants with lacunar stroke, compared with 5.8% (n = 8/137) of those with cortical stroke. Cardiac disease risk increased significantly with age (OR 1.04, 95% CI 1.019–1.081), male sex (female OR 0.36, 95% CI 0.201–0.676), and vascular risk factor score (OR 1.57, 95% CI 1.111–2.236) (Table 2 and Figure 2).

#### Vascular Risk Factors

**Figure 4:**
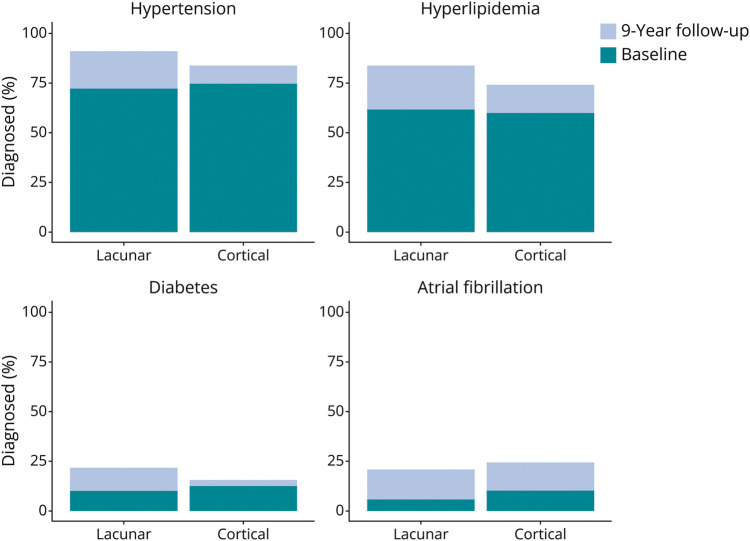
Participants With Diagnosed Hypertension, Diabetes, Hypercholesterolemia, and/or Atrial Fibrillation at Baseline, Along With New Diagnoses Acquired Between Baseline and the 9-Year Follow-Up, in Cases of Lacunar or Cortical Ischemic Stroke

At baseline, 72% (n = 76/106) of participants with lacunar stroke and 74% (n = 101/137) of those with cortical stroke had hypertension (χ^2^ = 0.042, *p* = 0.83). By 9 years, hypertension increased to 89% (n = 94/106) in participants with lacunar stroke and 83% (n = 114/137) in those with cortical stroke (χ^2^ = 1.039, *p* = 0.30) (Figure 4 and eTable3). Hypercholesterolemia was common in both lacunar and cortical stroke groups, at both baseline (62% [n = 66/106] vs 60% [n = 82/137]; χ^2^ = 0.062, *p* = 0.80) and 9 years (83% [n = 88/106] vs 74% [n = 101/137]; χ^2^ = 2.474, *p* = 0.11). Diabetes was more prevalent in the lacunar stroke group than in the cortical stroke group at 9 years (22% [n = 23/106] vs 15% [n = 21/137]; χ^2^ = 1.233, *p* = 0.26) although not at baseline (10.4% [n = 11/106] vs 12.4% [n = 17/137]; χ^2^ = 0.083, *p* = 0.77). Ten percent (n = 14/137) of participants with cortical stroke and 6% (n = 6/106) of those with lacunar stroke had atrial fibrillation at baseline (χ^2^ = 1.096, *p* = 0.29), increasing to 27% (n = 37/137) and 21% (n = 22/106) by follow-up (χ^2^ = 0.953, *p* = 0.32).

### Functional and Self-Reported Well-Being Outcomes

#### Disability and Dependence

**Figure 5:**
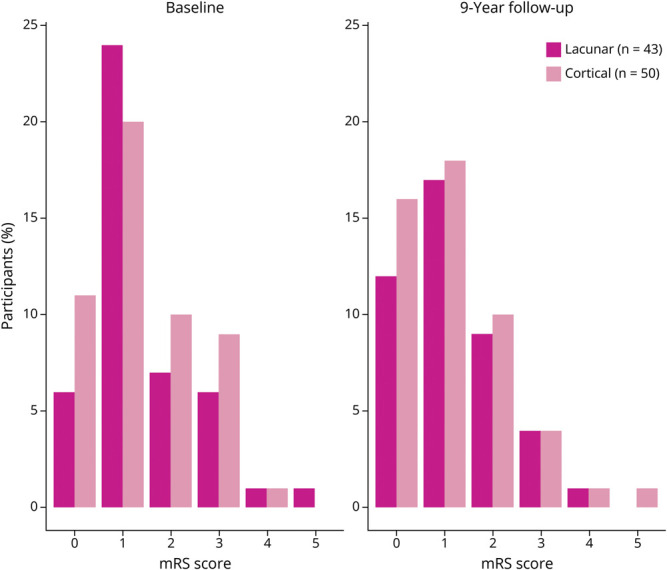
Longitudinal Disability and Functional Independence at Baseline and 9 Years After Minor Ischemic Lacunar (Dark Pink) or Cortical Stroke (Light Pink) (n = 93) mRS = modified Rankin Scale.

At baseline, 80% (n = 85/106) of participants with lacunar stroke and 77% (n = 105/137) of those with cortical stroke reported no to mild symptoms after stroke and were independent in activities of daily living (mRS scores 0–2). Moderate/severe disability (mRS scores 3–5) was reported in 20% (n = 21/106) of participants with lacunar stroke and 23% (n = 32/137) of those with cortical stroke. At 9 years, most remained independent (lacunar: 88% [n = 38/43]; cortical: 88% [n = 44/50]), while 12% (n = 5/43) of participants with lacunar stroke and 12% (n = 6/50) of those with cortical stroke reported moderate/severe disability (Figure 5).

For those who returned the postal questionnaire at 9 years (n = 96, 47% with lacunar), the median change in mRS score from baseline to 9 years was 0 (IQR −1 to 1). Between baseline and 9 years, 30.2% of participants with lacunar stroke and 28% of those with cortical stroke had a ≥1-point increase in the mRS score.

#### Activities of Daily Living and Self-Reported Well-Being

Most participants reported persisting problems from their index stroke (lacunar: 38% [n = 17/45]; cortical: 45% [n = 22/49]; χ^2^ = 1.39, *p* = 0.49) (Table 3 and eFigures 2 and 3).

**Table 3:** Self-Reported Functional, Neuropsychiatric, and Well-Being Outcomes at 9 Years After Lacunar or Mild Cortical Ischemic Stroke (n = 96)

	Lacunar (N = 45)	Cortical (N = 51)	Overall (N = 96)	Result (*p* value)
Age, mean ± SD	69 ± 7	72 ± 12	71 ± 10	−1.719, 7.477 (0.21)^a^
Sex, female, n (%)	20 (44)	18 (35)	38 (40)	—
Modified Rankin Scale score (n = 93), n (%)				1,012 (0.73)^b^
No symptoms at all	12 (27)	16 (31)	28 (29)	—
No significant disability	17 (38)	18 (35)	35 (36)	—
Slight disability	9 (20)	10 (20)	19 (20)	—
Moderate disability	4 (9)	4 (8)	8 (8)	—
Moderate-severe disability	1 (2)	1 (2)	2 (2)	—
Bedridden	0 (0)	1 (2)	1 (1)	—
Ongoing problems from stroke, yes, n (%)	17 (38)	22 (43)	39 (41)	1.399 (0.49)^c^
Subjective cognitive concerns, yes, n (%)	22 (49)	28 (55)	50 (52)	1.272 (0.52)^c^
Difficulty thinking quickly (n = 91), n (%)				859.5 (0.12)^b^
Not difficult	21 (47)	29 (57)	50 (52)	—
Somewhat difficult	12 (27)	12 (24)	24 (25)	—
Very difficult	11 (24)	6 (12)	17 (18)	—
Anxiety/depression (n = 94), n (%)				888.5 (0.06)^b^
None	23 (51)	33 (65)	56 (58)	—
Slight/moderate	8 (18)	9 (18)	17 (18)	—
Severe	14 (31)	7 (14)	21 (22)	—
Problems with self-care tasks (n = 93), n (%)				984.5 (0.28)^b^
None	34 (76)	41 (80)	75 (78)	—
Slight/moderate	11 (24)	5 (10)	16 (17)	—
Severe/unable	0 (0)	2 (4)	2 (2)	—
Difficulty performing household chores (n = 94), n (%)				966.5 (0.22)^b^
Not difficult	26 (58)	35 (69)	61 (64)	—
Somewhat difficult	17 (38)	11 (22)	28 (29)	—
Very difficult	2 (4)	3 (6)	5 (5)	—
How often are you limited socially? (n = 94), n (%)				996 (0.33)^b^
None of the time	27 (60)	34 (67)	61 (64)	—
A little of the time	14 (31)	12 (24)	26 (27)	—
All of the time	4 (9)	3 (6)	7 (7)	—
Mobility problems (n = 95), n (%)				1,136 (0.92)^b^
None	25 (56)	28 (55)	53 (55)	—
Slight/moderate	16 (36)	16 (31)	32 (33)	—
Severe/unable	4 (9)	6 (12)	10 (10)	—
Difficulty walking without losing balance (n = 94), n (%)				1,022.5 (0.48)^b^
Not difficult	26 (58)	32 (63)	58 (60)	—
Somewhat difficult	16 (36)	14 (27)	30 (31)	—
Very difficult	3 (7)	3 (6)	6 (6)	—
Regained strength of affected limb (n = 92), n (%)				705.5 (0.00)^b^
A lot of strength	14 (31)	26 (51)	40 (42)	—
Quite a bit of strength	13 (29)	15 (29)	28 (29)	—
A little strength	18 (40)	6 (12)	24 (25)	—
Problems with usual activities (n = 94), n (%)				1,123.5 (0.86)^b^
None	24 (53)	27 (53)	51 (53)	—
Slight/moderate	18 (40)	15 (29)	33 (34)	—
Severe/unable	3 (7)	7 (14)	10 (10)	—
Current housing arrangement (n = 92), n (%)				—
In a care home	1 (2)	1 (2)	2 (2)	—
On my own	14 (31)	13 (25)	27 (28)	—
With my partner or relatives	29 (64)	34 (67)	63 (66)	—

a Independent-samples *t* test (95% CI).

b Mann-Whitney *U* test (*W*).

c Chi-square (χ^2^).

While both lacunar (49% [n = 22/45]) and cortical (55% [n = 27/49]) stroke groups noted subjective cognitive concerns (χ^2^ = 1.272, *p* = 0.52), 52% (n = 23/44) of participants with lacunar stroke described *thinking quickly* as “somewhat” or “very” difficult, compared with only 38% (n = 18/47) of participants with cortical stroke (*W* = 859.5, *p* = 0.12) (Table 3).

Depression and anxiety were slightly more prevalent among participants with lacunar stroke (49% [n = 22/45] vs 33% [n = 16/49]) (χ^2^ = 4.014, *p* = 0.13). Of these, 31% (n = 14/45) of participants with lacunar stroke and 14% (n = 7/49) of those with cortical stroke rated their anxiety/depression symptoms as “severe” (*W* = 888.5, *p* = 0.06). Since their stroke, 40% (n = 18/45) of participants with lacunar stroke and 31% (n = 15/49) of those with cortical stroke reported feeling socially limited at least some of the time (*W* = 996, *p* = 0.33).

Difficulties with activities of daily living were common. 42% (n = 19/45) of participants with lacunar stroke reported difficulties completing household tasks, compared with 29% (n = 14/49) of those with cortical stroke (*W* = 966.5, *p* = 0.22). 24% (n = 11/45) of participants with lacunar stroke acknowledged facing challenges with self-care tasks, including washing and dressing, compared with 10% (n = 5/48) of participants with cortical stroke (*W* = 984.5, *p* = 0.28) (Table 3).

Problems with mobility, balance, and muscle strength were common: 36% (n = 16/45) of participants with lacunar stroke and 32% (n = 16/50) of those with cortical stroke had “slight-to-moderate” problems with mobility, and 9% (n = 4/45) of the lacunar stroke group and 12% (n = 6/50) of the cortical stroke group had severe mobility difficulties (*W* = 1,136, *p* = 0.92). Impaired balance was slightly more frequent in participants with lacunar stroke (42% [n = 19/45]) than in those with cortical stroke (35% [n = 17/49]) (*W* = 1,022.5, *p* = 0.48). Most participants with cortical stroke (55% [n = 26/47]) regained a substantial portion of strength in their affected limb after stroke, compared with 31% (n = 14/45) of those with lacunar strokes; a higher proportion of participants with lacunar stroke (40% [n = 18/45]) only gained a minimal amount of strength compared with 13% (n = 6/47) of those with cortical strokes (*W* = 705.5, *p* ≤ 0.001) (Table 3).

Most participants were living with family (lacunar: 66% [n = 29/44]; cortical: 71% [n = 34/48]), followed by those living independently (lacunar: 32% [n = 14/44]; cortical: 27% [n = 13/48]). Only 2% (n = 1/44) of lacunar and 2% (n = 1/48) of cortical stroke questionnaire respondents were residing in assisted living facilities.

## Discussion

We report longitudinal data on clinical, functional, and participant-reported outcomes 9 years after lacunar or mild cortical ischemic stroke. Despite the participants with lacunar strokes being younger at baseline, a similar proportion of the cortical and lacunar stroke groups developed dementia (11%), had a recurrent stroke (32%), or died (26%) by 9 years. A higher proportion of participants with cortical stroke died, particularly due to cardiovascular causes. Cardiac diagnoses, including IHD, arrhythmias, and heart failure, were more prevalent in the cortical stroke group. Conversely, hypertension, hypercholesterolemia, and diabetes were more frequent among participants with lacunar stroke. These profiles coincide with previous research results and likely reflect the differing vascular pathologies underlying the stroke subtypes: large artery atherothrombotic disease associated with cortical strokes and SVD characteristic of lacunar strokes.^8,21^ Notably, recurrent stroke was only associated with vascular risk factors in the first year after stroke, with less obvious association thereafter, despite recurrent stroke rates being similar between lacunar and cortical stroke groups.

Few studies have examined dementia risk in patients with lacunar stroke. Findings indicate that 11%–20% of patients develop dementia within 2–4 years after lacunar stroke, a proportion that increases to 15% after 9 years.^8,22-25^ In our study, 9% of participants with lacunar stroke had a dementia diagnosis. Despite being slightly younger, participants with lacunar stroke had dementia rates similar to those with cortical stroke. We may have underestimated dementia diagnoses because of several factors. Patients, their relatives, or primary health care providers may overlook or normalize early symptoms of dementia, perhaps attributing them to the aging process or perceiving a clinical diagnosis to be unnecessary, believing that it might not alter clinical management. Gradual symptom onset may also diminish their perceived significance and severity.

Lacunar strokes have a 22% recurrence rate within 5 years.^8,23,26,27^ In our study, 32% of participants had at least 1 recurrent stroke within 9 years, with a similar rate between cortical and lacunar stroke groups. While some studies suggest lower recurrence in lacunar strokes, recent findings challenge their favorable prognosis.^8^ In the context of SVD, we must also consider the role of clinically “silent” infarction, as in the case of most radiologically fleeting diffusion-weighted imaging–positive lesions, which are often clinically covert.^28,29^ Longitudinal SVD studies with frequent MRI neuroimaging are likely to provide valuable insights into the role of these lesions and their clinical outcome correlates.^28^ In our study, only vascular risk factors were associated with recurrent stroke risk, although previous studies found that SVD burden severity at baseline can play crucial roles in determining the likelihood of future stroke after lacunar stroke.^22^ Detailed etiologic classifications of recurrent strokes were not available within this data set, which limits interpretation of potential underlying mechanisms.

Death after lacunar stroke is infrequent in the first year but increases over time. Case fatality averages 2.8% at 1 year, rising to 27% after 5 years.^8^ In this study, 26% of participants had died by 9 years, with those with cortical stroke having worse survival. Longer term follow-up studies with relatively small samples of patients with lacunar stroke have shown that mortality rates can approach 60%–75% within 10–14 years after stroke.^26,27^ In our study, worse WMH burden at baseline was associated with higher risk of death. In addition to stroke and dementia, SVD progression is associated with higher risk of cognitive impairment and impaired mobility, which may lead to infections, hospitalizations, or falls.^30^ For stroke patients, cardiovascular deaths predominate in the short and long term. However, in this study, cardiovascular causes were the most common primary cause of death only among participants with cortical stroke.^8^

Compared with clinical outcomes such as recurrent stroke, global functional decline and quality of life have received far less attention as outcomes after lacunar stroke. Patients with lacunar stroke generally experience better functional outcomes compared to those with other stroke subtypes.^8,15,31^ However, dependency rates remain substantial, affecting over 40% of patients at 3 years after stroke.^24^ Over 10% of patients who maintain independence experience impairments in other areas, limiting social, occupational, or recreational participation.^8,24^ In our study, 40% of participants reported persistent effects from their initial stroke, with 12% reporting moderate/severe disability. Participants with lacunar stroke were more likely to experience poorer limb recovery and report problems with balance, psychiatric symptoms, and thinking speed.

A key strength of this study is its extended follow-up, particularly as similar studies rarely extend beyond 4 years.^8^ Other strengths include the use of multiple overlapping ascertainment methods, the inclusion of measures of functional status, and the high retention rate of participants. This was achieved in part by leveraging data collected directly from patient records, enabling long-term tracking. However, this also represents a limitation, due to potential missed patient diagnoses, particularly dementia. Our relatively small sample size may not fully reflect the broader aging SVD or cortical stroke patient population. The subset of participants who were able to complete the functional outcome questionnaire were younger, performed better in cognitive assessments, had a milder radiologic SVD burden at baseline, and were likely those with the lowest morbidity, which may affect the representativeness of our findings and understate the effects of progressing disease.

These findings suggest that long-term outcomes after minor stroke are often suboptimal and are influenced by stroke subtype pathophysiology. These findings also serve to highlight the considerable and enduring impact of lacunar and mild cortical ischemic stroke on quality of life. There is considerable scope for improving long-term outcomes in patients with mild ischemic stroke. Recognizing these long-term effects is essential for directing clinical management and guiding research priorities. Identifying possible early predictors of longitudinal SVD progression and unfavorable clinical and functional outcomes could be useful in clinical practice and aid in the development of prognostic tools.

## Glossary

ACE-R: Addenbrooke Cognitive Examination–Revised
GP: general practitioner
ICH: intracranial hemorrhage
ICV: intracranial volume
IHD: ischemic heart disease
mRS: modified Rankin Scale
NIHSS: NIH Stroke Scale
OR: odds ratio
RSSI: recent small subcortical infarct
SVD: small vessel disease
WMH: white matter hyperintensity
